# Risk of psychological ill health and methods of organisational downsizing: a cross-sectional survey in four European countries

**DOI:** 10.1186/s12889-017-4789-3

**Published:** 2017-09-29

**Authors:** Elena Andreeva, M. Harvey Brenner, Töres Theorell, Marcel Goldberg

**Affiliations:** 1 Centre for Applied Rehabilitation Research, Department of Rehabilitation Medicine, Hannover Medical School, Hannover, Germany; 2 Department of Behavioral and Community Health, School of Public Health, University of North Texas Health Science Center, Fort Worth, TX USA; 3 Department of Health Policy and Management, Johns Hopkins University Bloomberg School of Public Health, Baltimore, MD USA; 4 Institute for Stress Research, Stockholm University, Stockholm, Sweden; 5 Inserm, Population-based Epidemiologic Cohorts Unit, UMS, 11 Villejuif, France; 6 Paris Descartes University, Paris, France

**Keywords:** Reactive downsizing, Strategic downsizing, Unemployment, Surviving a layoff, Returning to work, Psychological ill health, Cross-sectional survey, European countries

## Abstract

**Background:**

The manner in which organizational downsizing is implemented can make a substantial difference as to whether the exposed workers will suffer from psychological ill health. Surprisingly, little research has directly investigated this issue. We examined the likelihood of psychological ill health associated with strategic and reactive downsizing.

**Methods:**

A cross-sectional survey included 1456 respondents from France, Sweden, Hungary and the United Kingdom: 681 employees in stable workplaces (reference group) and 775 workers from downsized companies. Reactive downsizing was exemplified by the exposures to compulsory redundancies of medium to large scale resulting in job loss or surviving a layoff while staying employed in downsized organizations. The workforce exposed to strategic downsizing was represented by surplus employees who were internally redeployed and supported through their career change process within a policy context of “no compulsory redundancy”. Symptoms of anxiety, depression and emotional exhaustion were assessed in telephone interviews with brief subscales from Hospital Anxiety Scale (HADS-A), Hopkins Symptom Checklist (SCL-CD_6_) and Maslach Burnout Inventory (MBI-GS). Data were analyzed using logistic regression.

**Results:**

We observed no increased risk of psychological ill health in the case of strategic downsizing. The number of significant associations with psychological ill health was the largest for the large-scale reactive downsizing: surviving a layoff was consistently associated with all three outcome measures; returning to work after the job loss experience was related to anxiety and depression, while persons still unemployed at interview had elevated odds of anxiety. After reactive medium-scale downsizing, unemployment at interview was the only exposure associated with anxiety and depression.

**Conclusions:**

The manner in which organizational downsizing is implemented can be important for the psychological wellbeing of workers. If downsizing is unavoidable, it should be achieved strategically. Greater attention is needed to employment and health policies supporting the workers after reactive downsizing.

**Electronic supplementary material:**

The online version of this article (10.1186/s12889-017-4789-3) contains supplementary material, which is available to authorized users.

## Background

Workforce downsizing, the reduction of personnel in organizations, was almost inevitable for survival of many companies during the Great Recession of the late 2000s. Yet, continued export of jobs to developing countries intrinsic to globalization, and technological developments improving productivity, are enduring sources of extensive downsizing in industrialized countries. Extensive downsizing frequently results in adverse consequences for workers’ health and psychological wellbeing [1]. However, empirical evidence of health risks related to downsizing remains inconsistent. Many studies found an increased likelihood of psychological ill health in laid off and remaining workers [2–6]. Less frequently, research documented null overall findings in persons who lost their jobs or remained employed in downsized organizations [7–9].

Previous research has largely failed to discriminate between different forms of downsizing that may lead to differential health risks in affected workers and represent a major source of inconsistent findings. To date at least two forms have been described in the literature. *Strategic downsizing* is often implemented without recourse to compulsory redundancies, mostly by means of reduced work hours, natural wastage, voluntary turnover and early retirement. It represents a planned approach aimed at promotion of long-term organizational benefits while minimizing negative individual impact [10]; organizations redeploy and retrain surplus workers in order to maintain skill levels. A contrasting form is *reactive downsizing* that exemplifies a response to short-term needs and external events, such as economic decline. Reactive downsizing is associated with compulsory redundancies and conducted without concern for process and outcome consistency with business strategy, mission and goals, or with requisite organizational culture and values [10]. Reactive downsizing is more common; most work has focused on this type and documented greater health risks after large-scale layoffs [11–14]. The research base on health effects of strategic downsizing is very small. We know of only one study which found no decrease in psychological wellbeing of employees from before to after strategic downsizing [15].

Therefore, with particular attention to the issue of downsizing strategies, the present research sought to examine the likelihood of psychological ill health associated with strategic and reactive downsizing. Reactive downsizing is exemplified by the exposures to compulsory redundancies of medium to large scale resulting in job loss or surviving a layoff while staying employed in the downsized organization. The workforce exposed to strategic downsizing is represented by surplus employees who were internally redeployed, retrained and supported through their career change process within a policy context of “no compulsory redundancy”, despite a considerable reduction in staffing. No attempts have been made so far to explicitly investigate the effects of both downsizing types in a single study, and there is very limited insight into the effects of internal redeployment in downsized firms. We therefore contribute to the literature by collecting empirical evidence on psychological health outcomes related to the different types of organizational downsizing. Our contribution addresses the idea of socially responsible restructuring proposed in theoretical and policy-oriented papers. This approach recognizes that people are the source of innovation and renewal; workers should be treated as assets to be developed rather than costs to be eliminated; companies can maintain their key competences if they retrain employees giving them a unique set of required skills. Companies should implement redundancies as a last resort, when other measures failed to secure expected financial viability, and consider the impact of downsizing on both leavers and stayers [16, 17].

Our main research hypotheses were formulated in advance. We assumed that the likelihood of psychological ill health will depend on downsizing strategy, employment status and extent of compulsory redundancies that occurred. Employee health and wellbeing will probably be less severely affected if their employers made clear efforts to proactively minimize job losses and maintain skill levels in the company while meeting organizational requirements. The study focusses on three outcomes including depressive symptoms, anxiety and emotional exhaustion.

## Methods

### Study sample

The cross-sectional Restructuring Survey was conducted in four European countries: France, Hungary, Sweden and the United Kingdom. The survey focused on the employees’ perception of organizational downsizing in relation to their health. Information was collected between April 2009 and mid-May 2011 on health outcomes and multiple dimensions of the downsizing process. The full details on the sample selection have been published elsewhere [18]. The study settings were chosen to account for different national models of protection from labor market risks. The conditions formed by these models are likely to influence labor market transitions, flexibility and security of employment. France, Hungary, Sweden and the UK represent, respectively, the Mediterranean-Continental, Eastern European, Nordic and Anglo-Saxon models. The study enrolled 1456 respondents: 681 employees who had never experienced downsizing and 775 workers from downsized companies. The downsized group included a sample of persons with one of the following changes in the employment status: (1) surviving a layoff while remaining employed in downsized organization, (2) occupational transition through internal redeployment, (3) job loss followed by unemployment and (4) reemployment. Two additional criteria defined this sample: recent downsizing during the last two years and workforce reductions of at least 10%. Excluded were workers exposed to non-recent or smaller-scale downsizing and employees who received a warn notice but retired, quit or found another job before becoming unemployed. In the non-downsized sample, excluded were nonworking persons with no experience of downsizing. Farmers, self-employed and workers of microenterprises with less than 10 employees were excluded from both downsized and non-downsized samples.

With the assumption of equally large downsized and non-downsized groups and with an expected symptom prevalence of 25% and 10%, respectively, we planned to include a minimum of 292 participants in each country for obtaining the results with statistical significance of alpha 0.05 (two-sided) and power 0.90. The calculation was adjusted upward up to a maximum of 400 respondents in each country in order to account for potential exclusion or nonresponse. Estimates of symptom prevalence were derived from occupational health studies [19, 20].

The sampling was carried out by using a targeted selection of respondents. We employed multiple sources to identify participants who meet the strict inclusion criteria: (1) prospective cohort studies based on nationally representative samples in Sweden (Swedish Longitudinal Occupational Survey of Health 2008/2010) [21, 22] and Hungary (Hungarostudy 2006) [23], (2) a random sample from the public telephone book in Hungary, (3) advertisement in a free newspaper with a nationwide circulation, recruitment through occupational physicians, health screening centers and public employment agencies in France and (4) a workforce sample from a large company in the United Kingdom. This company, the BT, announced job cuts of 10% of its workforce in 2009 and aimed to implement downsizing through natural wastage, non-replacement and voluntary redundancy [24]. The surplus permanent employees from restructured businesses were redeployed and retrained in a “transition center”. According to the statistics from the BT Group health adviser, 96% of such workers stay in BT after the redeployment. The BT employees were enrolled from the transition center, restructured and non-restructured business lines. The employer had no knowledge of who participated in the survey.

**Fig. 1:**
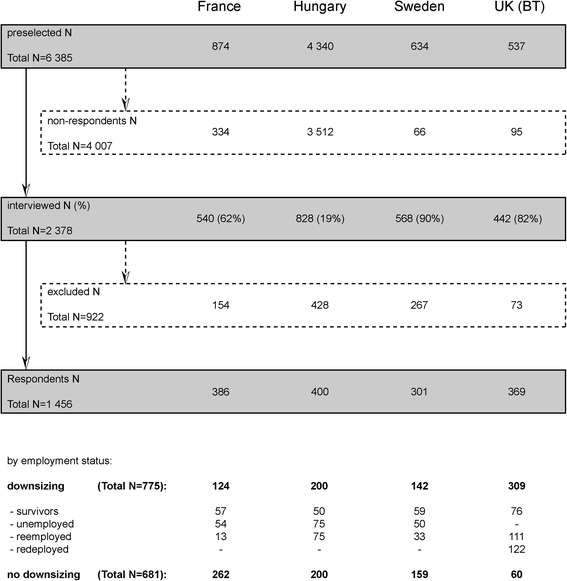
Flow chart: selection and participation in Restructuring Survey

The participation rates reached 90% in Sweden, 82% in the UK, 62% in France and 19% in Hungary. These rates take into account refusals and break-offs by respondents, non-interviews due to incorrect telephone numbers, as well as respondents’ never being available or being unavailable during the fieldwork. In the Hungarian sample, interviewers reported the highest rate of refusals (64%) and a high level of distrust, probably as a result of the socioeconomic situation during the recession of the late 2000s [25]. The sampling process is summarized in Fig. 1.

### Data and measures

#### Structured self-report interview

The survey respondents completed telephone interviews based on a structured questionnaire (Additional file 1 – Study Questionnaire). The questionnaire included demographic and health-related information for all participants and the section on downsizing for layoff survivors, redeployed, reemployed and unemployed.

#### Exposure to downsizing

Two items were used to assess downsizing in the respondent’s company, the question of whether the person has ever worked for employers who downsized significant number of staff and the question how the person was affected by this event. Employees who responded no on the first item were regarded as unexposed workers with no downsizing. This category included workers from non-downsized companies and the BT personnel from non-downsized business lines. The second item served for the classification of downsizing exposure in terms of altered *employment status* during the last two years. Workers who were laid off and became unemployed were further subdivided into unemployed and reemployed at interview, based on yes-or-no responses to the question “Have you got a new job yet?”. Persons who kept their jobs in the downsized organization were categorized as layoff survivors. The BT redeployees indicated having been currently in the transition center. Those BT workers who stayed in the transition center during the last two years, but had a new job or role at interview, were classified as reemployed. The rationale for considering employment status is based on earlier studies showing an increased risk of psychological ill health in laid off and remaining workers [2–6]. Our study extends the literature by including these groups from several countries and adding a new group of internally redeployed workers, which to our knowledge has never been compared to a reference group from non-downsized workplaces.

*Type and scale of downsizing* was classified into four categories: (1)” no downsizing”, (2)” strategic downsizing, no compulsory redundancies” in BT redeployees and reemployed workers who have been in the transition center, (3)” reactive medium scale downsizing with compulsory redundancies 10–19%” and (4)” reactive large-scale downsizing with compulsory redundancies ≥20%”.

We computed a composite variable for downsizing exposure with nine mutually exclusive categories denoting changes in employment status by type and scale of downsizing: 0 = “no downsizing” (reference group), 1 = “strategic downsizing, redeployed”, 2 = “strategic downsizing, reemployed”, 3 = “reactive medium-scale downsizing, reemployed”, 4 = “reactive medium-scale downsizing, layoff survivor”, 5 = “reactive medium-scale downsizing, unemployed”, 6 = “reactive large-scale downsizing, reemployed”, 7 = “reactive large-scale downsizing, layoff survivor”, 8 = “reactive large-scale downsizing, unemployed”. Codes 1 and 2 refer to BT personnel who were, respectively, redeployed in the transition center at interview or already completed their redeployment and received a new job or role in BT. The approach of building a composite variable was suggested by Rothman [26] and used in occupational epidemiology to study the odds ratio patterns for all meaningful combinations of exposures [27].

#### Outcomes

The brief version of the Hopkins Symptom Checklist 90 (SCL-90) is a 6-item scale (SCL-CD_6_) covering the core *symptoms of depression* [28, 29]. Participants were asked how much they have been troubled by each symptom over the prior week. Responses were “not at all”, “a little”, “moderately”, “quite a bit” and “very much” yielding a total score from 0 to 24. The SCL-CD_6_ scale was examined before with reference to the DSM-IV diagnosis of major depression; scores of 17 or greater were predictive of subsequent antidepressant use and hospitalisations for depressive episodes [30]. Hence, we defined the score ≥ 17 as indicative of elevated symptom level and dichotomized the variable at this cut point (high versus low level of symptoms). The high level of symptoms is not equivalent to a clinical diagnosis of major depression, but it indicates an increased depression risk.

The 7-item Hospital Anxiety Scale (HADS-A) measured symptoms associated with anxiety [31]. Respondents rated how often they experienced each symptom on a five-point Likert scale from “never” to “always”. We classified the subjects according to their total score (from 0 to 28) as being likely to have high (≥15) versus low level of *anxiety symptoms* (<15). With the scores dichotomized at the upper sextile of the non-downsized group, the proportions of high-level symptoms in our respondents (12.5% in the non-downsized group, 20.1% in the total sample) were very close to the prevalence estimates from meta-analysis [32] and studies which used HADS-A in general populations or occupational settings [33, 34]. In this study, HADS-A was administered in telephone interviews with a modified response format. Methods of administration were found to significantly affect the reports of anxiety with HADS-A [35]. Recall of response categories can be a source of bias in telephone surveys. In order to minimize recall bias in the absence of visual cues, we used the same format for rating the symptom frequency with HADS-A and the Maslach Burnout Inventory, as these scales followed each other in interviews.

*Emotional exhaustion* was measured with the 5-item subscale from the Maslach Burnout Inventory (MBI-GS) [36]. The response scale indicates the frequency of experiencing each symptom on a five-point Likert scale from “never” to “always”. A total score was calculated by adding the points obtained on each item divided by five [37]. For the analysis, we converted the total score into dichotomous categories, high versus low level of symptoms, by placing the cut-off at the upper tertile of the non-downsized group. This strategy for defining the cut-off takes into account previous epidemiological findings based on MBI-GS [37, 38]: the prevalence rates of high-level symptoms in our subjects are comparable to those reported before, i.e. 32.2% in the non-downsized group and 38.6% in the total sample.

Additional file 2 shows an overview of all study instruments applied for measuring health.

#### Covariates: Demographic characteristics and health behaviors

Age in years, gender and education (university or equivalent degree versus any lower education) were considered as potential confounders. Dummy variables denoting the country of respondents’ residence were created to account for unobservable country-specific effects due to national differences in social protection, health systems and flexibility of labor markets. Health behaviors included smoking (daily and occasional smokers versus current non-smokers) and frequency of alcohol drinking coded as 1 = “never” (abstainers, reference group), 2 = “once a month or less”, 3 = “2–4 times a month”, 4 = “2–3 times a week”, 5=“4 times a week or more”.

### Statistical analysis

We compared characteristics of study participants by using the χ2 test or analysis of variance, when appropriate. In the next step, we applied multivariate logistic regression to explore the associations between downsizing exposures and outcomes including depressive symptoms, anxiety and emotional exhaustion. We investigated the effects of exposures in terms of a composite variable reflecting changes in employment status by type and scale of downsizing. This approach aims at providing risk estimates in greater detail than before by considering the effects of strategic [15] and reactive downsizing, scale of redundancies [12–14, 39] and altered employment status [5, 6] in multiple groups of workers. In all logistic regression analyses, we used workers with no downsizing as the reference group. We reported fully and partially adjusted odds ratios (OR) with 95% confidence intervals (95% CI). Partially adjusted models include exposure status as the main predictor plus country-specific effects entered as covariates. Fully adjusted models were estimated by entering, in addition, age, gender, education, smoking and frequency of alcohol drinking. Exposure status and frequency of alcohol drinking were treated as factor variables: this procedure creates dummy variables for the levels of categorical regressors [40]. For all analyses, we used the STATA software package, version SE 11.2 for Windows. Significance was considered at *p* < 0.05.

## Results

### Descriptive statistics

Table 1 provides an overview of the study population. Additional file 3 shows demographic characteristics and health behaviors by exposure status.

**Table 1:** Characteristics of study participants, *n* (%) or mean (SD)

Characteristic	Respondents (*N* = 1456)
Age: years	Mean = 45.0 (SD = 10.3) Range = 18–68
Sex	
Men	790 (54.3)
Women	666 (45.7)
Education	
University	654 (44.9)
Any lower education	802 (55.1)
Country	
Hungary	400 (27.5)
Sweden	301 (20.7)
France	386 (26.5)
UK	369 (25.3)
Smoking	
Daily or occasional smoker	341 (23.4)
Non-smoker	1115 (76.6)
Frequency of alcohol drinking	
“never” (abstainer)	170 (11.7)
“once a month or less”	315 (21.6)
“2–4 times a month”	466 (32.0)
“2–3 times a week”	346 (23.8)
“4 times a week or more”	157 (10.8)
“don’t know” (non-abstainer)	2 (0.1)
Downsizing exposure	
Workers with no downsizing	681 (46.8)
Strategic downsizing (no CR): redeployed	122 (8.4)
Strategic downsizing (no CR): reemployed	111 (7.6)
Reactive medium-scale downsizing (CR 10–19%): reemployed	73 (5.0)
Reactive medium-scale downsizing (CR 10–19%): survivor	98 (6.7)
Reactive medium-scale downsizing (CR 10–19%): unemployed	109 (7.5)
Reactive large-scale downsizing (CR ≥20%): reemployed	44 (3.0)
Reactive large-scale downsizing (CR ≥20%): survivor	126 (8.7)
Reactive large-scale downsizing (CR ≥20%): unemployed	67 (4.6)
Reactive downsizing, scale unknown	25 (1.7)
Depressive symptoms	
Missing or incomplete data	34 (2.3)
Sum score	Mean = 6.1 (SD = 5.6) Range = 0–24
Anxiety	
Missing or incomplete data	41 (2.8)
Sum score	Mean 10.4 (SD = 5.4) Range = 0–28
Emotional exhaustion	
Missing or incomplete data	161 (11)
Sum score	Mean = 1.5 (SD = 0.9) Range = 0–4

*Abbreviations*: *N* number of respondents in the sample, *n* number of participants in respective categories, *(%)* percent, *SD* standard deviation, *CR* presence and extent of compulsory redundancies measured as percent reduction in personnel

Of those exposed to any type of downsizing, the majority were men (59.1%; 458) and persons without university education (59.6%; 462). In the non-downsized group, both sexes were almost equal in proportion, and a half of respondents reported university education. A total of 1244 (85%) out of 1456 participants responded to all items on mental health and well-being. The largest partial non-response was observed for emotional exhaustion (11.1%; 161), due to the high rate of missingness in the Swedish sample (43.9%; 132): difficulties were experienced with the item “how often would a full day at work be really taxing for you?”, probably because of the subjunctive mood in the wording. This grammar form, in Swedish termed *Konjunktiv*, is becoming increasingly rare in Standard Swedish. For the analyses, missing values on a taxing day at work were replaced for Swedish participants with their mean values on the remaining items of MBI-GS. After this replacement, the proportion of non-missing values on all outcomes increased to 93.5% (1362) in all respondents and to 95% (286) in the Swedish sample.

Table 2 shows the prevalence of mental health conditions. The results indicate the presence of statistically significant country-specific effects: all outcomes were more prevalent in participants from the UK and Hungary (all *p* < 0.01). We also found significant differences by exposure status. Persons reemployed after reactive medium-scale downsizing and workers of the non-downsized organizations can be regarded as the healthiest groups, as reflected by the lowest prevalence of all symptoms. Emotional exhaustion occurred most frequently in layoff survivors and persons with strategic downsizing (*p* < 0.001). The prevalence of depression and anxiety were higher in reemployed workers after large-scale downsizing (*p* < 0.001).

**Table 2:** Prevalence of psychological ill health by socio-demographic covariates, health behaviours and downsizing exposure

Characteristic	Depressive symptoms	Anxiety	Emotional exhaustion
	high level n/N(%)	high level n/N(%)	high level n/N(%)
Total sample	89 / 1422 (6.3)	285 / 1415 (20.1)	547 / 1416 (38.6)
Age group	*p = 0.073*	*p = 0.268*	*p = 0.417*
18 to 34 years	13 / 267 (4.9)	44 / 261 (16.9)	94 / 265 (35.5)
35 to 49 years	48 / 602 (8.0)	122 / 607 (20.1)	242 / 602 (40.2)
50 years and over	28 / 553 (5.1)	119 / 547 (21.8)	211 / 549 (38.4)
Gender	***p = 0.019***	***p = 0.026***	*p = 0.090*
Men	38 / 778 (4.9)	139 / 773 (18.0)	282 / 770 (36.6)
Women	51 / 644 (7.9)	146 / 642 (22.7)	265 / 646 (41.0)
Education	*p = 0.795*	*p = 0.863*	*p = 0.589*
University	39 / 642 (6.1)	130 / 639 (20.3)	251 / 637 (39.4)
Any lower education	50 / 780 (6.4)	155 / 776 (20.0)	296 / 779 (38.0)
Country	***p = 0.003***	***p < 0.001***	***p < 0.001***
Hungary	31 / 374 (8.3)	80 / 370 (21.6)	129 / 380 (34.0)
Sweden	7 / 299 (2.3)	24 / 295 (8.1)	98 / 290 (33.8)
France	20 / 381 (5.3)	66 / 383 (17.2)	117 / 377 (31.0)
UK	31 / 368 (8.4)	115 / 367 (31.3)	203 / 369 (55.0)
Smoking	*p = 0.057*	*p = 0.943*	*p = 0.689*
Daily or occasional smoker	28 / 330 (8.5)	64 / 320 (20.0)	124 / 329 (37.7)
Non-smoker	61 / 1092 (5.6)	221 / 1095 (20.2)	423 / 1087 (38.9)
Frequency of alcohol drinking	***p = 0.021***	*p = 0.078*	*p = 0.249*
“never” (abstainer)	19 / 164 (11.6)	46 / 163 (28.2)	71 / 163 (43.6)
“once a month or less”	17 / 300 (5.7)	59 / 302 (19.5)	117 / 299 (39.1)
“2–4 times a month”	20 / 457 (4.4)	80 / 453 (17.7)	159 / 457 (34.8)
“2–3 times a week”	19 / 343 (5.5)	65 / 342 (19.0)	131 / 341 (38.4)
“4 times a week or more”	14 / 156 (9.0)	35 / 153 (22.9)	68 / 154 (44.2)
“don’t know” (non-abstainer)	0 / 2 (0.0)	0 / 2 (0.0)	1 / 2 (50.0)
Downsizing exposure	***p < 0.001***	***p < 0.001***	***p < 0.001***
Workers with no downsizing	27 / 664 (4.1)	82 / 658 (12.5)	212 / 659 (32.2)
Strategic downsizing (no CR): redeployed	6 / 121 (5.0)	35 / 121 (28.9)	60 / 122 (49.2)
Strategic downsizing (no CR): reemployed	7 / 111 (6.3)	31 / 111 (27.9)	58 / 111 (52.2)
Reactive medium-scale downsizing (CR 10–19%): reemployed	1 / 69 (1.4)	8 / 72 (11.1)	17 / 70 (24.3)
Reactive medium-scale downsizing (CR 10–19%): survivor	7 / 98 (7.1)	21 / 96 (21.9)	43 / 96 (44.8)
Reactive medium-scale downsizing (CR 10–19%): unemployed	12 / 106 (11.3)	33 / 107 (30.8)	34 / 104 (32.7)
Reactive large-scale downsizing (CR ≥20%): reemployed	8 / 41 (19.5)	15 / 40 (37.5)	18 / 40 (45.0)
Reactive large-scale downsizing (CR ≥20%): survivor	15 / 124 (12.1)	31 / 122 (25.4)	68 / 125 (54.4)
Reactive large-scale downsizing (CR ≥20%): unemployed	3 / 64 (4.7)	19 / 63 (30.2)	26 / 65 (40.0)
Reactive downsizing, scale unknown	3 / 24 (12.5)	10 / 25 (40.0)	11 / 24 (45.8)

*Note*: These analyses are restricted to respondents with complete data on psychological ill health symptoms. In the Swedish sample, prevalence of emotional exhaustion was calculated after handling the partial non-response on one MBI-GS item

*Abbreviations*: *n* number of respondents with mental health conditions, *N* number of participants with complete responses, *(%)* prevalence in percent, *CR* presence and extent of compulsory redundancies measured as percent reduction in personnel

*p* values for Pearson’s χ2 test of between-group differences (in bold type: considered statistically significant if p < 0.05)

### Multivariate logistic regression analysis

Table 3 displays the associations between the downsizing exposures and mental health conditions. The results on *strategic downsizing* indicate no evidence that being redeployed or reemployed in BT was related to increased risks of psychological ill health. The fully adjusted results on *reactive downsizing* showed that the likelihood of emotional exhaustion was significantly increased only in survivors of large-scale layoffs (OR = 2.04, *p* < 0.01). Reemployment after the large-scale downsizing was strongly associated with a roughly fourfold greater odds of scoring poorly on the scale of anxiety (*p* < 0.001) and depressive symptoms (*p* < 0.01). Elevated odds of suffering from high level symptoms of depression (OR = 2.78, *p* < 0.01) and anxiety (OR = 1.77, *p* < 0.05) were also observed in survivors of large-scale layoffs. Unemployment was significantly related to anxiety in workers who lost jobs through medium- and large-scale downsizing, with a roughly fourfold increase in odds of high level symptoms (all *p* < 0.001). Association with depression was significant in unemployed persons after medium- (OR = 3.42, *p* < 0.01) but not large-scale redundancies.

**Table 3:** Odds ratios and 95% confidence intervals for the associations between downsizing exposure and mental health conditions

		^a^Model 1			^b^Model 2		
		*N*	OR (95% CI)	*p* value	*N*	OR (95% CI)	*p* value
Depressive symptoms							
Type of downsizing	Employment status						
No downsizing	workers/no downsizing	664	1 (ref.)		664	1 (ref.)	
Strategic	redeployed	121	0.56 (0.19 to 1.61)	0.282	121	0.54 (0.18 to 1.60)	0.268
	reemployed	111	0.72 (0.26 to 2.00)	0.529	109	0.72 (0.26 to 2.04)	0.539
Reactive medium-scale	reemployed	69	0.38 (0.05 to 2.92)	0.355	69	0.38 (0.05 to 2.90)	0.348
	survivors	98	1.53 (0.62 to 3.79)	0.358	98	1.54 (0.62 to 3.83)	0.357
	unemployed	106	3.48 (1.67 to 7.24)	0.001	106	3.42 (1.63 to 7.20)	0.001
Reactive large-scale	reemployed	41	4.98 (2.01 to 12.31)	0.001	41	3.79 (1.48 to 9.69)	0.005
	survivors	124	2.55 (1.26 to 5.16)	0.009	124	2.87 (1.39 to 5.92)	0.004
	unemployed	64	1.17 (0.34 to 4.03)	0.807	64	1.11 (0.32 to 3.87)	0.872
Anxiety							
Type of downsizing	Employment status						
No downsizing	workers/no downsizing	658	1 (ref.)		658	1 (ref.)	
Strategic	redeployed	121	0.94 (0.52 to 1.69)	0.838	121	0.86 (0.47 to 1.56)	0.609
	reemployed	111	0.90 (0.49 to 1.63)	0.720	109	0.85 (0.46 to 1.57)	0.603
Reactive medium-scale	reemployed	72	1.08 (0.49 to 2.40)	0.843	72	1.11 (0.50 to 2.49)	0.792
	survivors	96	1.50 (0.84 to 2.69)	0.171	96	1.55 (0.86 to 2.78)	0.145
	unemployed	107	3.97 (2.43 to 6.47)	<0.001	107	4.19 (2.54 to 6.91)	<0.001
Reactive large-scale	reemployed	40	4.31 (2.12 to 8.78)	<0.001	40	4.20 (2.03 to 8.70)	<0.001
	survivors	122	1.76 (1.06 to 2.91)	0.029	122	1.77 (1.06 to 2.97)	0.030
	unemployed	63	3.50 (1.91 to 6.42)	<0.001	63	3.81 (2.06 to 7.05)	<0.001
Emotional exhaustion							
Type of downsizing	Employment status						
No downsizing	workers/no downsizing	659	1 (ref.)		659	1 (ref.)	
Strategic	redeployed	122	0.73 (0.43 to 1.24)	0.245	122	0.71 (0.42 to 1.22)	0.220
	reemployed	111	0.83 (0.48 to 1.42)	0.488	109	0.80 (0.46 to 1.39)	0.430
Reactive medium-scale	reemployed	70	0.73 (0.41 to 1.31)	0.289	70	0.75 (0.42 to 1.36)	0.348
	survivors	96	1.29 (0.81 to 2.04)	0.284	96	1.30 (0.81 to 2.07)	0.274
	unemployed	104	1.14 (0.73 to 1.77)	0.573	104	1.18 (0.75 to 1.86)	0.467
Reactive large-scale	reemployed	40	1.88 (0.98 to 3.64)	0.059	40	1.77 (0.91 to 3.46)	0.092
	survivors	125	1.96 (1.31 to 2.95)	0.001	125	2.04 (1.35 to 3.08)	0.001
	unemployed	65	1.55 (0.91 to 2.63)	0.104	65	1.50 (0.88 to 2.56)	0.139

Results from multivariate logistic regression analysis

*Abbreviations*: *N* number of respondents, *OR* odds ratio, *95% CI* 95% confidence interval, *ref.* reference group

^a^Model 1: adjusted for country-specific effects

^b^Model 2: Model 1 + adjusted for demographic data (age, sex, education) and health behaviors (smoking and frequency of alcohol drinking)

## Discussion

The aim of this study was to analyze the odds of psychological ill health in workers exposed to recent downsizing, as compared to employees in stable workplaces. In extension to previous research, we considered the exposures to both strategic and reactive downsizing in a large multi-country sample. Our findings suggest that the manner in which downsizing is undertaken makes a substantial difference as to whether the exposed workers will suffer from symptoms of depression, anxiety or emotional exhaustion. In the case of *strategic downsizing*, we observed no increased risk of psychological ill health. This result is well in line with an earlier study in a British chemical processing plant which implemented strategic downsizing without negative consequences for psychological health of employees; the company put a heavy emphasis on retraining and redeploying staff to maintain skill levels and minimize job losses [15]. Our earlier analysis of a smaller data set showed that the majority of redeployees reported maintenance of income level, skill upgrading and other help by employers for smoothing the transition to a new employment [18]. We can therefore assume considerable savings in health costs and maintenance of worker capacity if downsizing is implemented strategically.

Further results imply that *reactive downsizing* involving compulsory redundancies produced adverse effects. The number of significant associations with psychological ill health was the largest for the *large-scale downsizing*: surviving a large-scale layoff was consistently associated with all three outcome measures; unemployment at interview was related to anxiety, while reemployed persons had elevated odds of anxiety and depression symptoms. Our finding of an increased burden of all examined symptoms in survivors of large-scale layoffs concurs with much of the recent research [5, 12–14, 39, 41]. Past analyses suggest that adverse effects of surviving a layoff result from multiple stressors: depletion of energetic resources (e.g., due to increased workload) [27, 39, 42, 43], disrupted personal goals (e.g., decreased training opportunities) [43], destabilization of the psychosocial climate at work [1] and continuous job insecurity due to repeated rounds of downsizing [39, 44]. The exposure to large-scale layoffs during the Great Recession has probably made the group of remaining employees exceptionally vulnerable to the stress of potential reactive downsizing.

Our data showed inconsistent patterns in reemployed workers exposed to reactive downsizing. The finding that the likelihood of suffering from psychological ill health is not increased in those who returned to work after *medium-scale redundancies* is in line with the recent studies [45]. Going back to work after the job loss experience may provide a complete or partial reversal of the adverse health effects of unemployment [4, 45]. However, we failed to find beneficial effects of returning to work after *large-scale redundancies*. Specifically, in workers who lost jobs due to large-scale layoffs, the likelihood of high-level anxiety symptoms was similar in reemployed and still unemployed persons, while the burden of depressive symptoms was significantly increased only in reemployed. This is consistent with recent work showing the medium- and long-term effects of unemployment in the Great Recession on stress-related cardiovascular illness and self-perceived health [46–48]. The mixed evidence can also be attributed to a number of methodological reasons. First, because of the cross-sectional nature of our data, we could not control for health conditions prior to downsizing and thus for the directionality of the associations. In particular, ill health can act as a barrier to gaining a high quality reemployment characterized by job security and good working conditions [49]. In turn, poor job quality following reemployment may act as a barrier to improved health [49] or even as a risk factor for increases in minor psychiatric morbidity [50]. Second, factors determining reemployment success – that is, finding work quickly and/or finding a good job [51] – were beyond the scope of our Restructuring Survey. Thus, we could not control for the effects of delayed reemployment or underemployment in new jobs. Other studies which have data on these factors documented poorer mental health after delayed reemployment [52] and in persons reemployed with fewer working hours, using fewer skills and receiving less pay than they could if they were working at full capacity [53]. A high prevalence of delayed reemployment and underemployment may therefore be a possible explanation for the increased burden of anxiety and depression symptoms in our respondents who returned to work after large-scale redundancies. Finally, it is important to interpret our results in the broader macroeconomic context that highlights the link between large-scale layoffs, quality/speed of reemployment and psychological ill health. The risk of unemployment and a job seeker’s reemployment success depend on the labor market’s need for employees. It is likely that our respondents who returned to work after large-scale redundancies were previously employed in industries severely affected by recession. Due to constraints of the labor market, these workers were particularly disadvantaged in terms of reemployment success and thus suffered emotional distress.

Further results of our study imply that the extent of reactive downsizing – the company’s economic context in which a person became unemployed – may influence the strength of association between current unemployment and depressive symptoms. Unemployment due to medium-scale layoffs was strongly related to depression. However, we did not find evidence that the likelihood of symptoms was increased in persons still unemployed after large-scale redundancies. This finding is in line with earlier studies at individual level suggesting that the adverse health effects of unemployment are less prominent when unemployment increases in the general population [54, 55]. This may be attributed to health selection factors: probably, the workers who lost jobs due to medium-scale downsizing were primarily those with pre-existing depression. Thus, the impact of health selection on subsequent unemployment has been found in a Finnish study using panel data [56]. When layoffs are massive, the increased numbers of symptom-free people may also become unemployed. As a result, the odds ratios for depressive symptoms in the group of unemployed due to large-scale redundancies may become considerably attenuated. It is also possible that the stigma of losing a job is reduced when layoffs are massive [57].

We consider the distinction between strategic and reactive downsizing as a strength, because contrasting both types of downsizing in a single study has earlier been neglected. Our findings should however be interpreted in light of some limitations. One important drawback includes the cross-sectional design precluding causal inferences. Second, due to self-report nature of our data, the results are susceptible to common method bias. Yet we believe that we could reduce the risk of this bias by assuring total anonymity and relying on voluntary participation. Third, symptoms of psychological ill health were not validated by a physician. Thus, when our respondents scored poorly on the scales of depression, anxiety or emotional exhaustion, this did not necessarily reflect the presence of a clinically significant disease. However, the scales (SCL-CD_6_, HADS-A and MBI-GS) have earlier been found to perform well in measuring the increased risk of symptoms in epidemiological research [30, 31, 36], and our results should thus be interpreted in relation to increased risk of psychological ill health. Finally, a potential for selection bias should be considered. Refusals and nonresponse could have been particularly prevalent among migrants with insufficient language skills or the working poor without telephone lines; these groups could have an increased risk of downsizing and decreased mental health. It is therefore possible that the strength of the associations observed in this study is attenuated.

We feel it reasonable to believe that our results are representative in terms of variation in downsizing strategies in European organizations during the Great Recession of the late 2000s. However, the results are not necessarily generalizable across time and outside the European Union. Furthermore, given our research focus on labor market participants who could not withdraw from their downsizing situations, the results are not necessarily generalizable to all groups of European workers. We excluded persons who chose withdrawal through early retirement or found another job before becoming unemployed. These exposures might be associated with either poorer or better health. Older age, poor health and difficulties of finding new employment may influence the decision to retire early. In contrast, obtaining new employment before the actual job loss might by and large be more frequent among younger, healthier and better educated workers. Further research should investigate the generalizability of findings in these groups of workers.

## Conclusions

This multi-country study adds new information to the identification of differences in psychological health of workers exposed to strategic and reactive downsizing. The manner in which organizational downsizing is implemented can be important for the psychological wellbeing of workers. The results have implications for workforce planning: if downsizing is unavoidable, it should ideally be achieved in a strategic manner, without compulsory redundancies. Our findings also imply the need for greater attention to employment and health policies aimed at supporting workers with a recent experience of reactive downsizing. In particular, socially responsible restructuring should incorporate strategies of primary and secondary prevention focused on monitoring and improvement of workers’ psychological health. This seems to be of particular relevance for survivors and newly reemployed workers after large-scale layoffs. Employers and health professionals should create a comprehensive program to minimize the negative impact of layoffs on health of workers.

## Additional files

Additional file 1: Study Questionnaire – English Version. (PDF 552 kb)

Additional file 2: Study instruments for measuring health and wellbeing. (DOC 54 kb)

Additional file 3: Demographic characteristics and health behaviors of study participants by detailed exposure status (*N* = 1456). (DOC 91 kb)

## Abbreviations

CI: Confidence interval
CR: Compulsory redundancies
HADS-A: Hospital Anxiety and Depression Scale – Anxiety subscale
MBI-GS: Maslach Burnout Inventory – General Survey
OR: Odds Ratio
ref.: reference group
SCL-CD_6_: Symptom Checklist – core depression scale, 6 items
SD: Standard deviation
UK: United Kingdom

## References

[1] Noer D (1993). Healing the wounds: overcoming the trauma of layoffs and revitalizing downsized organizations.

[2] Brand JE, Levy BR, Gallo WT (2008). Effects of layoffs and plant closings on depression among older workers. Res Aging.

[3] Mandal B, Roe B (2008). Job loss, retirement and the mental health of older Americans. J Ment Health Policy Econ.

[4] Gallo WT, Bradley EH, Siegel M, Kasl SV (2000). Health effects of involuntary job loss among older workers: findings from the health and retirement survey. J Gerontol B Psychol Sci Soc Sci.

[5] Kivimäki M, Honkonen T, Wahlbeck K, Elovainio M, Pentti J, Klaukka T (2007). Organisational downsizing and increased use of psychotropic drugs among employees who remain in employment. J Epidemiol Community Health.

[6] Moore S, Grunberg L, Greenberg E (2004). Repeated downsizing contact: the effects of similar and dissimilar layoff experiences on work and well-being outcomes. J Occup Health Psychol.

[7] Breslin FC, Mustard C (2003). Factors influencing the impact of unemployment on mental health among young and older adults in a longitudinal, population-based survey. Scand J Work Environ Health.

[8] Østhus S (2012). Health effects of downsizing survival and job loss in Norway. Soc Sci Med.

[9] Martikainen P, Mäki N, Jäntti M (2008). The effects of workplace downsizing on cause-specific mortality: a register-based follow-up study of Finnish men and women remaining in employment. J Epidemiol Community Health.

[10] Kozlowski SWJ, Chao GT, Smith EM, Hedlund J (1993). Organizational downsizing: strategies, interventions, and research implications. Int Rev Ind Organ Psychol.

[11] Westgaard RH, Winkel J (2011). Occupational musculoskeletal and mental health: Significance of rationalization and opportunities to create sustainable production systems - A systematic review. Appl Ergon.

[12] Vahtera J, Kivimäki M, Pentti J, Linna A, Virtanen M, Virtanen P (2004). Organisational downsizing, sickness absence, and mortality: 10-town prospective cohort study. BMJ.

[13] Vahtera J, Kivimäki M, Pentti J (1997). Effect of organisational downsizing on health of employees. Lancet.

[14] Vahtera J, Kivimäki M, Forma P, Wikström J, Halmeenmäki T, Linna A (2005). Organisational downsizing as a predictor of disability pension: the 10-town prospective cohort study. J Epidemiol Community Health.

[15] Parker SK, Chmiel N, Wall TD (1997). Work characteristics and employee well-being within a context of strategic downsizing. J Occup Health Psychol.

[16] Cascio WF (2005). Strategies for responsible restructuring. Akad Manage Perspect.

[17] Hansen GB (2009). A guide to worker displacement: Some tools for reducing the impact on workers, communities and enterprises.

[18] Brenner MH, Andreeva E, Theorell T, Goldberg M, Westerlund H, Leineweber C (2014). Organizational downsizing and depressive symptoms in the European recession: the experience of workers in France, Hungary, Sweden and the United Kingdom. PLoS One.

[19] Jenkins R, Harvey S, Butler T, Thomas RL (1996). Minor psychiatric morbidity, its prevalence and outcome in a cohort of civil servants – a seven-year follow-up study. Occup Med (Lond).

[20] Sanderson K, Nicholson J, Graves N, Tilse E, Oldenburg B (2008). Mental health in the workplace: using the ICF to model the prospective associations between symptoms, activities, participation and environmental factors. Disabil Rehabil.

[21] Magnusson Hanson LL, Theorell T, Bech P, Rugulies R, Burr H, Hyde M (2009). Psychosocial working conditions and depressive symptoms among Swedish employees. Int Arch Occup Environ Health.

[22] Leineweber C, Baltzer M, Magnusson Hanson LL, Westerlund H (2013). Work-family conflict and health in Swedish working women and men: a 2-year prospective analysis (the SLOSH study). Eur J Pub Health.

[23] Susánszky É, Székely A, Szabó G, Szántó Zs, Klinger A, Konkolÿ Thege B, et al. A Hungarostudy Egészség Panel (HEP) felmérés módszertani leírása. [Methodological description of the Hungarian epidemiological panel (HEP) survey]. Mentálhigiéné és Pszichoszomatika. 2007;8(4):259-276. Hungarian.

[24] Jones A, Evans G. BT doubles job cuts to 30,000. The Independent. Thursday 14 May 2009. http://www.independent.co.uk/news/business/news/bt-doubles-job-cuts-to-30000-1684727.html. Accessed 16 Dec 2016.

[25] Horváth B (2010). On the nature of nonresponse and interviewer effects in the Hungarian Labour Force Survey. Hungarian Statistical Review.

[26] Greenland S, Rothman KJ, Rothman KJ, Greenland S (1998). Concepts of interaction. Modern epidemiology.

[27] Dragano N, Verde PE, Siegrist J (2005). Organisational downsizing and work stress: testing synergistic health effects in employed men and women. J Epidemiol Community Health.

[28] Derogatis LR, Rickels K, Rock AF (1976). The SCL-90 and the MMPI: a step in the validation of a new self-report scale. Br J Psychiatry.

[29] Lipman RS, Sartorius N, Ban TA (1986). Depression scales derived from Hopkins Symptom Checklist. Assessment of depression.

[30] Magnusson Hanson LL, Westerlund H, Leineweber C, Rugulies R, Osika W, Theorell T (2014). The Symptom Checklist-core depression (SCL-CD6) scale: psychometric properties of a brief six item scale for the assessment of depression. Scand J Public Health.

[31] Zigmond AS, Snaith RP (1983). The hospital anxiety and depression scale. Acta Psychiatr Scand.

[32] Baxter AJ, Scott KM, Vos T, Whiteford HA (2013). Global prevalence of anxiety disorders: a systematic review and meta-regression. Psychol Med.

[33] Lisspers J, Nygren A, Söderman E (1997). Hospital Anxiety and Depression Scale (HAD): some psychometric data for a Swedish sample. Acta Psychiatr Scand.

[34] Kleppa E, Sanne B, Tell GS (2008). Working overtime is associated with anxiety and depression: the Hordaland Health Study. J Occup Environ Med.

[35] Jörngården A, Wettergen L, von Essen L (2006). Measuring health-related quality of life in adolescents and young adults: Swedish normative data for the SF-36 and the HADS, and the influence of age, gender, and method of administration. Health Qual Life Outcomes.

[36] Schaufeli WB, Leiter MP, Maslach C, Jackson SE, Maslach C, Jackson SE, Leiter MP (1996). Maslach Burnout Inventory-General Survey. The Maslach Burnout Inventory-Test manual.

[37] Kowalski C, Driller E, Ernstmann N, Alich S, Karbach U, Ommen O (2010). Associations between emotional exhaustion, social capital, workload, and latitude in decision-making among professionals working with people with disabilities. Res Dev Disabil.

[38] Lasalvia A, Bonetto C, Bertani M, Bissoli S, Cristofalo D, Marrella G (2009). Influence of perceived organisational factors on job burnout: survey of community mental health staff. Br J Psychiatry.

[39] Kivimäki M, Vahtera J, Pentti J, Ferrie JE (2000). Factors underlying the effect of organisational downsizing on health of employees: longitudinal cohort study. BMJ.

[40] StataCorp. Working with categorical data and factor variables. In: [U] Stata User’s Guide: Release 11. College Station, TX: Stata Press; 2009. p. 339–356.

[41] Kalimo R, Taris TW, Schaufeli WB (2003). The effects of past and anticipated future downsizing on survivor well-being: an equity perspective. J Occup Health Psychol.

[42] Sigursteinsdóttir H, Rafnsdóttir GL (2015). Sickness and sickness absence of remaining employees in a time of economic crisis: a study among employees of municipalities in Iceland. Soc Sci Med.

[43] Jones MD, Sliter M, Sinclair RR (2016). Overload, and cutbacks, and freezes, oh my! The relative effects of the recession-related stressors on employee strain and job satisfaction. Stress Health.

[44] Ferrie JE, Shipley MJ, Stansfeld SA, Marmot MG (2002). Effects of chronic job insecurity and change in job security on self-reported health, minor psychiatric morbidity, physiological measures, and health related behaviours in British civil servants: the Whitehall II study. J Epidemiol Community Health.

[45] Rueda S, Chambers L, Wilson M, Mustard C, Rourke SB, Bayoumi A (2012). Association of returning to work with better health in working-aged adults: a systematic review. Am J Public Health.

[46] Jacob JA (2016). Greater health care spending may moderate recession's negative health effects. JAMA.

[47] Brenner MH. The impact of unemployment on heart disease and stroke mortality in European Union countries. Brussels: European Commission, Employment, Social Affairs and Inclusion; 2016 May. Report No.: KE-02-16-631-EN-N. Contract No.: #VT/2013/112 and #VT/2014/079. http://ec.europa.eu/social/BlobServlet?docId=15932&langId=en. Accessed 16 Feb 2017.

[48] Brenner MH. Duration of unemployment and self-perceived health in Europe. European Commission, Employment, Social Affairs and Inclusion; 2016 Mar. Report No.: KE-04-16-541-EN-N. Contract No.: #VT/2015/035. http://ec.europa.eu/social/BlobServlet?docId=15931&langId=en. Accessed 26 Feb 2017.

[49] Leach LS, Butterworth P, Strazdins L, Rodgers B, Broom DH, Olesen SC (2010). The limitations of employment as a tool for social inclusion. BMC Public Health.

[50] Ferrie JE, Martikainen P, Shipley MJ, Marmot MG, Stansfeld SA, Smith GD (2001). Employment status and health after privatisation in white collar civil servants: prospective cohort study. BMJ.

[51] Wanberg CR (2012). The individual experience of unemployment. Annu Rev Psychol.

[52] Ginexi EM, Howe GW, Caplan RD (2000). Depression and control beliefs in relation to reemployment: what are the directions of effect?. J Occup Health Psychol.

[53] Monfort SS, Howe GW, Nettles CD, Weihs KL (2015). A longitudinal examination of re-employment quality on internalizing symptoms and job-search intentions. J Occup Health Psychol.

[54] Martikainen PT, Valkonen T (1996). Excess mortality of unemployed men and women during a period of rapidly increasing unemployment. Lancet.

[55] Martikainen P, Mäki N, Jäntti M (2007). The effects of unemployment on mortality following workplace downsizing and workplace closure: a register-based follow-up study of Finnish men and women during economic boom and recession. Am J Epidemiol.

[56] Böckerman P, Ilmakunnas P (2009). Unemployment and self-assessed health: evidence from panel data. Health Econ.

[57] Clark A (2003). Unemployment as a Social Norm: Psychological Evidence from Panel Data. J Labor Econ.

