# Supplementary material for: Risk of psychological ill health and methods of organisational downsizing: a cross-sectional survey in four European countries
Source: BMC Public Health. 2017 Sep 29;17:758. doi: 10.1186/s12889-017-4789-3 (PMC5622469; doi:10.1186/s12889-017-4789-3)
Supplement: Supplementary file 1 — Study Questionnaire – English Version. (PDF 552 kb) [file 12889_2017_4789_MOESM1_ESM.pdf]

STUDY QUESTIONNAIRE
ENGLISH VERSION

Potential users of this questionnaire (which is used by courtesy of the European Commission) should contact the authors.

1. Are you or were you ever working for employers who have shed significant number of staff (ie "downsized")?

1 ☐ YES
2 ☐ NO → GO TO Q4

2. If yes, did this happen during <u>last two years?</u>

1 ☐ YES
2 ☐ NO → STOP THE INTERVIEW

3. Were you self-employed or a farmer, or worked in an organisation with less than 10 employees before the downsizing?

1 ☐ YES → STOP THE INTERVIEW
2 ☐ NO → GO TO Q5

4. Are you self-employed or a farmer, or work in an organisation with less than 10 employees?

1 ☐ YES → STOP THE INTERVIEW
2 ☐ NO, I AM NOT WORKING → STOP THE INTERVIEW
3 ☐ NO, I AM EMPLOYED IN AN ORGANIZATION WITH MORE THAN 10 EMPLOYEES → GO TO Q22

5. How large a proportion of the employees were made redundant during downsizing? Was it …

1 ☐ … **less than 10 %** → STOP THE INTERVIEW
2 ☐ … **between 10 % and 20 % or**
3 ☐ … **20 % or more?**
9 ☐ DON'T KNOW

6. Now I want you to tell me which of the following statements best describes how you were affected by the downsizing:

Read the numbers before the statements.

1 ☐ **1 - You were laid off and became unemployed**
2 ☐ **2 - You were notified, but found another job before becoming unemployed** → STOP THE INTERVIEW
3 ☐ **3 - You were notified, but never actually laid off, and stayed in employment in the same organisation** → GO TO Q12
4 ☐ **4 - You were notified and took early retirement** → STOP THE INTERVIEW
5 ☐ 5 **– Something else**

7. If you were laid off and became unemployed, for how long (including periods of multiple unemployment) within the last two years:

1 ☐ **less then three months**
2 ☐ **between three and six months**
3 ☐ **between six months and one year**
4 ☐ **one year or longer**

8. a) Have you got a new job yet?

1 ☐ YES
2 ☐ NO

9. a) Was financial compensation offered in the case of your losing your job?

1 ☐ YES
2 ☐ NO → GO TO Q10
9 ☐ DON'T KNOW → GO TO Q10

b) Was it an offer of a one-time payment, an arrangement corresponding to a salary during a defined period (e.g. for three month) or was it something else?

1 ☐ ONE-TIME PAYMENT → GO TO Q10
2 ☐ SALARY DURING A DEFINED PERIOD → GO TO Q10
3 ☐ SOMETHING ELSE

c) What kind of financial compensation?

……………………………………………………………………………………

10. Now, tell me about the job conditions you had before the downsizing

		USUALLY 1	SOMETIMES 2	NEVER 3	DON'T KNOW 9
a.	**Did you have enough time to do your work tasks: was it usually, sometimes or never?**	☐	☐	☐	☐
b.	**Did your work often involve conflicting demands: was it usually, sometimes or never?**	☐	☐	☐	☐
c.	**Did you have the possibility of learning new things through your work?** *If necessary read response categories*	☐	☐	☐	☐
d.	**Did you have a choice in deciding what to do in your work?**	☐	☐	☐	☐
e.	**Did have a choice in deciding how you do your work?**	☐	☐	☐	☐

11. a) Were you offered re-training to increase your likelihood of getting a new job?

1 ☐ YES
2 ☐ NO
9 ☐ DON'T KNOW / NOT APPLICABLE

b) Did you receive any other type of help by your company to prepare yourself to find new work?

1 ☐ YES
2 ☐ NO
9 ☐ DON'T KNOW / NOT APPLICABLE

12. **a) Did your employer give you notification in advance regarding the plans for downsizing?**

1 ☐ YES
2 ☐ NO → GO TO Q14

b) What type of notification? Was it …

1 ☐ …**in a personal meeting with your boss**
2 ☐ …**by e-mail or telephone**
3 ☐ … **during a general meeting**
4 ☐ … **by rumour?**
9 ☐ CAN'T REMEMBER

13. **How long time before the redundancy was the first notification from the employer given?**

Wait for a spontaneous answer. If necessary read response categories.

1 ☐ A WEEK OR LESS
2 ☐ BETWEEN A WEEK AND A MONTH
3 ☐ BETWEEN ONE AND THREE MONTHS
4 ☐ BETWEEN THREE AND SIX MONTHS
5 ☐ LONGER THAN SIX MONTHS
9 ☐ CAN'T REMEMBER

14. **And now I will ask some questions about how you would describe the process of downsizing. Especially, I want you to think about how decisions were made, no matter if you liked the outcome or not.**

Would you describe the process of downsizing to be …

		YES	NO	DON'T KNOW
		1	2	9
a.	… **transparent and understandable?**	☐	☐	☐
b.	… **fair and unbiased?**	☐	☐	☐
c.	… **chaotic?**	☐	☐	☐
d.	… **well planned?**	☐	☐	☐
e.	… **democratic?**	☐	☐	☐

15. **Do you agree that it was necessary to downsize the organisation:**

1 ☐ YES
2 ☐ NO
9 ☐ DON'T KNOW

16. Were you a manager responsible for staff?

1 ☐ YES
2 ☐ NO → GO TO Q18

17. Were you yourself forced to layoff personnel?

1 ☐ YES
2 ☐ NO

18. Did you feel that you could influence how the downsizing was carried out:

1 ☐ YES
2 ☐ NO
9 ☐ DON'T KNOW

19. a) What was the main reason for downsizing stated by the employer? Was it …

1 ☐ …**lack of work tasks**
2 ☐ …**financial cuts**
3 ☐ …**something else?**
9 ☐ CAN'T REMEMBER

b) Did you think the main reason stated by employer was the true motive?

1 ☐ YES
2 ☐ NO
9 ☐ DON'T KNOW

c) Did personal factors influence which employees were dismissed?

1 ☐ YES
2 ☐ NO
9 ☐ DON'T KNOW

20. After downsizing, how would you describe your organisation?
Would you describe it as …

		YES 1	NO 2	DON'T KNOW 9
a.	… **more efficient?**	☐	☐	☐
b.	… **understaffed?**	☐	☐	☐
c.	… **well organised?**	☐	☐	☐
d.	… **chaotic?**	☐	☐	☐
e.	… **insecure?**	☐	☐	☐

21. **Is your current level of income and benefits..?**

1 ☐ **equal to or higher than before the downsizing**

2 ☐ **lower than before the downsizing**

3 ☐ **substantially lower than before the downsizing**

22. *Skip if person is unemployed!*

Now I will ask you about the job conditions you had during the past month.

		USUALLY 1	SOMETIMES 2	NEVER 3	DON'T KNOW 9
a.	**Do you have enough time to do your work tasks: is it usually, sometimes or never?**	☐	☐	☐	☐
b.	**Does your work often involve conflicting demands: is it usually, sometimes or never?**	☐	☐	☐	☐
c.	**Do you have the possibility of learning new things through your work?** *If necessary read response categories*	☐	☐	☐	☐
d.	**Do you have a choice in deciding what you do in your work?**	☐	☐	☐	☐
e.	**Do you have a choice in deciding how you do your work?**	☐	☐	☐	☐

This concludes the first part of the interview. The rest of the interview will be questions related to your health.

23. Would you rate your general state of health to be …

1 ☐ … **very bad**
2 ☐ … **quite bad**
3 ☐ … **neither good nor bad**
4 ☐ … **quite good or**
5 ☐ … **very good?**

24. In general how happy do you consider yourself to be on a scale from 1 to 5 where 1 means not happy and 5 means very happy?

1 ☐ NOT HAPPY
2 ☐
3 ☐
4 ☐
5 ☐ VERY HAPPY

25. In a difficult situation, how often can you count on help from your …

		USUALLY<br>1	SOMETIMES<br>2	NEVER<br>3	DON'T KNOW<br>9
a.	… **neighbours: is it usually, sometimes or never?**	☐	☐	☐	☐
b.	… **co-workers: is it usually, sometimes or never?**	☐	☐	☐	☐
c.	… **family and relatives?** *If necessary read response categories.*	☐	☐	☐	☐
d.	… **friends?**	☐	☐	☐	☐
e.	… **spouse or partner?**	☐	☐	☐	☐

26. Next, I will ask you some questions about feelings and emotions that can arise. Please answer from 1 to 5 where 1 expresses "Never" and 5 refers to "Always"

How often …

	NEVER	RARELY	SOME-TIMES	OFTEN	ALWAYS	DON'T KNOW
	1	2	3	4	5	9
a. … **do you feel tense or "wound up"?** *If necessary repeat: "answer from 1 to 5 where 1 means "Never" and 5 means "Always"".*	☐	☐	☐	☐	☐	☐
b. … **do you get a sort of frightened feeling as if something awful is about to happen?**	☐	☐	☐	☐	☐	☐
c. … **do you have worrying thoughts go through your mind?**	☐	☐	☐	☐	☐	☐
d. … **can you sit at ease and feel relaxed?**	☐	☐	☐	☐	☐	☐
e. … **do you feel restless as if you have to be on the move?**	☐	☐	☐	☐	☐	☐
f. … **do you get sudden feelings of panic?**	☐	☐	☐	☐	☐	☐
g. **…do you get a sort of frightened feeling like 'butterflies' in your stomach?**	☐	☐	☐	☐	☐	☐

27. How often …

	NEVER	RARELY	SOME-TIMES	OFTEN	ALWAYS	DON'T KNOW
	1	2	3	4	5	9
a. … **do you feel emotionally drained:** *If necessary repeat: "answer from 1 to 5 where 1 means "Never" and 5 means "Always"".*	☐	☐	☐	☐	☐	☐
b. … **do you feel completely worn out at the end of the day?**	☐	☐	☐	☐	☐	☐
c. … **do you feel tired when you get up in the morning to meet a new day?**	☐	☐	☐	☐	☐	☐
d. … **would a full day at work be really taxing for you?**	☐	☐	☐	☐	☐	☐
e. … **do you feel burned out?**	☐	☐	☐	☐	☐	☐

28. Now I will ask you some questions about how you have felt in the past week. Please answer from 1 to 5 where 1 expresses "Not at all" and 5 refers to "Very much"

How much have you been troubled by …

		NOT AT ALL	A LITTLE	MODERATELY	QUITE A BIT	VERY MUCH	DON'T KNOW
		1	2	3	4	5	9
a.	… **lethargy or a lack of energy?** *If necessary repeat: "answer from 1 to 5 where 1 expresses "Not at all" and 5 refers to "Very much""*.	☐	☐	☐	☐	☐	☐
b.	… **feeling blue?**	☐	☐	☐	☐	☐	☐
c.	… **blaming yourself?**	☐	☐	☐	☐	☐	☐
d.	… **worrying too much?**	☐	☐	☐	☐	☐	☐
e.	… **feeling no interests in things?**	☐	☐	☐	☐	☐	☐
f.	… **a feeling that everything is an effort?**	☐	☐	☐	☐	☐	☐

29. **a) Do you think you get enough sleep and I want you to answer on a scale from 1 to 5 where 1 means definitely and 5 means definitely not?**

1 ☐ DEFINITELY
2 ☐
3 ☐
4 ☐
5 ☐ DEFINITELY NOT
9 ☐ DON'T KNOW

b) Besides sleep, do you think you get adequate time for rest and relaxation?

If necessary read: on a scale from 1 to 5 where 1 means definitely and 5 means definitely not?

1 ☐ DEFINITELY
2 ☐
3 ☐
4 ☐
5 ☐ DEFINITELY NOT
9 ☐ DON'T KNOW

c) Do you have enough time for yourself?

If necessary read: on a scale from 1 to 5 where 1 means definitely and 5 means definitely not?

1 ☐ DEFINITELY
2 ☐
3 ☐
4 ☐
5 ☐ DEFINITELY NOT
9 ☐ DON'T KNOW

30. **All things considered, do you sleep …**

1 ☐ … **very well**
2 ☐ … **quite well**
3 ☐ … **neither well nor badly**
4 ☐ … **quite badly or**
5 ☐ … **very badly?**
9 ☐ DON'T KNOW

31. **All in all, how satisfied are your with your life in general on a scale from 1 to 7 where 1 means very dissatisfied and 7 means very satisfied?**

1 ☐ VERY DISSATISFIED
2 ☐
3 ☐
4 ☐
5 ☐
6 ☐
7 ☐ VERY SATISFIED
9 ☐ DON'T KNOW

And now some questions about tobacco and alcohol.

32. a) Do you smoke everyday?

1 ☐ YES → GO TO Q33
2 ☐ NO

b) Do you smoke occasionally?

1 ☐ YES → GO TO Q34
3 ☐ NO

33. a) How many cigarettes a day do you smoke on average?

☐☐ 9 ☐ DON'T KNOW

34. How often do you drink alcohol?

Wait for a spontaneous answer. If necessary read response categories.

1 ☐ NEVER → GO TO Q36
2 ☐ ONCE A MONTH OR LESS
3 ☐ 2-4 TIMES A MONTH
4 ☐ 2-3 TIMES A WEEK
5 ☐ 4 TIMES A WEEK OR MORE
9 ☐ DON'T KNOW

35. How often do you drink six glasses or more on the same occasion? Is it …

1 ☐ **… never**
2 ☐ **… less than once a month**
3 ☐ **… every month**
4 ☐ **… every week or**
5 ☐ **... almost daily?**
9 ☐ DON'T KNOW

And now finally some questions about you personally

36. *Please indicate the respondent's sex*

1 ☐ MALE
2 ☐ FEMALE

37. What is your age?

☐☐ YEARS

38. Do you have university education or equivalent degree (Bachelors, Diploma, Masters etc)?

1 ☐ YES
2 ☐ NO

39. What is the final level of your education?

1 ☐ LESS THAN 8 YRS (BELOW LOWER SECONDARY, NO VOCATIONAL EDUCATION)
2 ☐ 9-10 YRS (LOWER SECONDARY, NO VOCATIONAL EDUCATION)
3 ☐ 11-12 YRS (LOWER SECONDARY PLUS VOCATIONAL SCHOOL)
4 ☐ 13-16 YRS (UPPER SECONDARY, VOCATIONAL COLLEGE)
5 ☐ UNIVERSITY LEVEL OR EQUIVALENT (E.G. UNIVERSITY OF APPLIED SCIENCES)
6 ☐ DOCTORAL DEGREE

40. What is or was your occupation?

☐☐☐☐☐☐☐☐☐☐☐☐☐☐☐☐☐☐☐☐

Thank you for participating!
