# Supplementary material for: Risk of psychological ill health and methods of organisational downsizing: a cross-sectional survey in four European countries
Source: BMC Public Health. 2017 Sep 29;17:758. doi: 10.1186/s12889-017-4789-3 (PMC5622469; doi:10.1186/s12889-017-4789-3)
Supplement: Supplementary file 2 — Study instruments for measuring health and wellbeing. (DOC 54 kb) [file 12889_2017_4789_MOESM2_ESM.doc]

**Additional file 2.** Study instruments for measuring health and wellbeing

| **Instrument** | **Wording** | **Variable codes and definitions** |
| --- | --- | --- |
| **SCL-CD_6_:**  Depression subscale of the Hopkins Symptom Checklist 90 (SCL-90), brief version | *How much during the last week have you been troubled by… (a) lethargy or lack of energy?  (b) feeling blue?  (c) blaming yourself?  (d) worrying too much? (e) feeling no interest in things?  (f) a feeling that everything is an effort?* | Level of depressive symptoms: 0=“low level” (scores 0-16);  1=”high level” (scores 17-24) |
| **HADS-A:** Hospital Anxiety Scale | *How often… (a) do you feel tense or “wound up”?  (b) do you get a sort of frightened feeling as if something awful is about to happen?  (c) do you have worrying thoughts go through your mind?  (d) can you sit at ease and feel relaxed?  (e) do you feel restless as if you have to be on the move?  (f) do you get sudden feelings of panic?  (g) do you get a sort of frightened feeling like ‘butterflies’ in your stomach?* | Level of anxiety symptoms: 0=“low level” (scores 0-14); 1=”high level” (scores 15-28) |
| **MBI-GS:**  Maslach Burnout Inventory General Survey | *How often… (a) do you feel emotionally drained?  (b) do you feel completely worn out at the end of the day?  (c) do you feel tired when you get up in the morning to meet a new day?  (d) would a full day at work be really taxing for you?  (e) do you feel burned out?* | Emotional exhaustion - symptom level: 0=”low level” (scores 0-1.6); 1=”high level” (scores 1.7-4) |
