# Supplementary material for: Risk of psychological ill health and methods of organisational downsizing: a cross-sectional survey in four European countries
Source: BMC Public Health. 2017 Sep 29;17:758. doi: 10.1186/s12889-017-4789-3 (PMC5622469; doi:10.1186/s12889-017-4789-3)
Supplement: Supplementary file 3 — Demographic characteristics and health behaviors of study participants by detailed exposure status (N = 1456). (DOC 91 kb) [file 12889_2017_4789_MOESM3_ESM.doc]

**Additional file 3. Demographic characteristics and health behaviors of study participants by detailed exposure status (N=1456)**

| **Characteristic** | **no downsizing** | **strategic downsizing (no compulsory redundancies)** | | **reactive downsizing, medium-scale (compulsory redundancies 10-19%)** | | | **reactive downsizing, large-scale (compulsory redundancies ≥20%)** | | | *** reactive downsizing, scale unknown** | **p value** |
| --- | --- | --- | --- | --- | --- | --- | --- | --- | --- | --- | --- |
|  |  | **redeployed** | **reemployed** | **reemployed** | **survivor** | **unemployed** | **reemployed** | **survivor** | **unemployed** |  |  |
|  | **n=681 (100)** | **n=122 (100)** | **n=111 (100)** | **n=73 (100)** | **n=98 (100)** | **n=109 (100)** | **n=44 (100)** | **n=126 (100)** | **n=67 (100)** | **n=25 (100)** |  |
| Age: years |  |  |  |  |  |  |  |  |  |  | <0.001† |
| mean ± SD | 43.9 ± 10.7 | 50.7 ± 5.6 | 49.5 ± 6.8 | 43.0 ± 11.5 | 45.7 ± 9.2 | 43.9 ± 10.7 | 41.1 ± 9.9 | 46.2 ± 10.4 | 41.2 ± 11.0 | 43.8 ± 9.2 |  |
| range | 18 - 68 | 34 - 61 | 21 - 64 | 20 - 63 | 24 - 63 | 21 - 65 | 19 - 59 | 21 - 64 | 21 - 63 | 26 - 59 |  |
| Sex |  |  |  |  |  |  |  |  |  |  | <0.001 |
| men | 332 (48.8) | 85 (69.7) | 81 (73.0) | 39 (53.4) | 61 (62.2) | 63 (57.8) | 13 (29.6) | 76 (60.3) | 33 (49.3) | 7 (28.0) |  |
| women | 349 (51.2) | 37 (30.3) | 30 (27.0) | 34 (46.6) | 37 (37.8) | 46 (42.2) | 31 (70.4) | 50 (39.7) | 34 (50.7) | 18 (72.0) |  |
| Education |  |  |  |  |  |  |  |  |  |  | <0.001 |
| university | 341 (50.1) | 41 (33.6) | 41 (36.9) | 23 (31.5) | 36 (36.7) | 37 (33.9) | 17 (38.6) | 68 (54.0) | 32 (47.8) | 18 (72.0) |  |
| any lower education | 340 (49.9) | 81 (66.4) | 70 (63.1) | 50 (68.5) | 62 (63.3) | 72 (66.1) | 27 (61.4) | 58 (46.0) | 35 (52.2) | 7 (28.0) |  |
| Country |  |  |  |  |  |  |  |  |  |  | <0.001 |
| Hungary | 200 (29.4) |  |  | 38 (52.0) | 15 (15.3) | 40 (36.7) | 34 (77.3) | 32 (25.4) | 34 (50.7) | 7 (28.0) |  |
| Sweden | 159 (23.3) |  |  | 28 (38.4) | 32 (32.7) | 34 (31.2) | 5 (11.4) | 27 (21.4) | 16 (23.9) |  |  |
| France | 262 (38.5) |  |  | 7 (9.6) | 19 (19.4) | 35 (32.1) | 5 (11.4) | 26 (20.6) | 17 (25.4) | 15 (60.0) |  |
| UK | 60 (8.8) | 122 (100) | 111 (100) |  | 32 (32.6) |  |  | 41 (32.5) |  | 3 (12.0) |  |
| Smoking |  |  |  |  |  |  |  |  |  |  | <0.001 |
| daily or occasional smoker | 171 (25.1) | 9 (7.4) | 10 (9.0) | 21 (28.8) | 20 (20.4) | 40 (36.7) | 15 (34.1) | 20 (15.9) | 28 (41.8) | 7 (28.0) |  |
| non-smoker | 510 (74.9) | 113 (92.6) | 101 (91.0) | 52 (71.2) | 78 (79.6) | 69 (63.3) | 29 (65.9) | 106 (84.1) | 39 (58.2) | 18 (72.0) |  |
| Frequency of alcohol drinking |  |  |  |  |  |  |  |  |  |  | <0.001 |
| “never” (abstainer) | 75 (11.0) | 16 (13.1) | 15 (13.5) | 7 (9.6) | 11 (11.2) | 17 (15.6) | 12 (27.3) | 6 (4.8) | 8 (11.9) | 3 (12.0) |  |
| “once a month or less” | 146 (21.4) | 11 (9.0) | 13 (11.7) | 23 (31.5) | 19 (19.4) | 29 (26.6) | 11 (25.0) | 32 (25.4) | 23 (34.3) | 8 (32.0) |  |
| “2-4 times a month” | 256 (37.6) | 24 (19.7) | 23 (20.7) | 31 (42.5) | 30 (30.6) | 36 (33.0) | 8 (18.2) | 36 (28.6) | 15 (22.4) | 7 (28.0) |  |
| “2-3 times a week” | 144 (21.2) | 46 (37.7) | 39 (35.1) | 9 (12.3) | 25 (25.5) | 20 (18.4) | 7 (15.9) | 39 (30.9) | 14 (20.9) | 3 (12.0) |  |
| “4 times a week or more” | 60 (8.8) | 25 (20.5) | 19 (17.1) | 3 (4.1) | 13 (13.3) | 7 (6.4) | 6 (13.6) | 13 (10.3) | 7 (10.4) | 4 (16.0) |  |
| “don’t know” (non-abstainer) |  |  | 2 (1.8) |  |  |  |  |  |  |  |  |

Abbreviations: n, number of participants in respective categories; (%) percent; SD, standard deviation.

* reactive downsizing, scale unknown: Of 25 persons in this group, 18 (72%) were layoff survivors, 4 (16%) reemployed and 3 (12%) unemployed

† p values for the analysis of variance of between-group differences

p values for Pearson’s χ2 test of between-group differences
